# The effect of COVID-19 on mental well-being in Switzerland: a cross-sectional survey of the adult Swiss general population

**DOI:** 10.1186/s12875-021-01532-7

**Published:** 2021-09-10

**Authors:** Laura Diaz Hernandez, Stéphanie Giezendanner, Roland Fischer, Andreas Zeller

**Affiliations:** Centre for Primary Health Care, University of Basel, Kantonsspital Baselland, Rheinstrasse 26, 4410 Liestal, Switzerland

**Keywords:** COVID-19, Outbreak, Coronavirus, Mental health, SARS-CoV-2

## Abstract

**Background:**

In addition to the threat of the COVID-19 pandemic to physical health, mental health is challenged by the emotional response to the situation and the official measures taken to stop the pandemic. This study aimed to assess the prevalence of impaired mental well-being due to COVID-19 and explore associated factors.

**Methods:**

The study was an observational, population-based, nationwide, cross-sectional online survey of a representative sample of the general Swiss population performed between March and April 2020. Participants reported on mental well-being, self-isolation/quarantine, their risk for developing severe COVID-19, and their work situation. Multivariable logistic regression analyses assessed risk factors for impaired mental well-being due to the pandemic.

**Results:**

Data from 1022 individuals were analysed. The median age was 44 years (range 18 to 78) and 49% were women. A third of respondents reported that the COVID-19 pandemic impaired their mental well-being and almost half reported specific mental health concerns. Impaired mental well-being was associated with having health problems (OR = 1.88, 95% CI: 1.29–2.74, vs no problems), being or living with someone at risk for severe COVID-19 (OR = 1.38, 95% CI: 1–1.9,), smoking (OR = 1.8, 95% CI: 1.24–2.61), living in urban residential environments (OR = 1.62, 95% CI: 1.13–2.32, vs rural), not being able to work due to closed workplace (OR = 1.66, 95% CI: 1.04–2.67), aged between 18 and 29 years old (OR = 1.99, 95% CI: 1.32–3.01, vs 45 to 59 years old), and living in a single household (living with someone, OR = 0.65, 95% CI: 0.44–0.97,vs single household). Overall, the most significant covariates of impaired mental well-being were specific mental health concerns: feeling depressed (OR = 7.21, 95% CI: 4.5–11.55), feeling less pleasure in doing things than before (OR = 6.28, 95% CI: 4.1–9.62), feeling anxious (OR = 6.13, 95% CI: 3.91–9.59) and feeling lonely (OR = 4.08, 95% CI: 2.53–6.58).

**Conclusion:**

Impaired mental well-being can carry long-term consequences. We encourage policymakers to implement strategies to promote mental health during this pandemic situation. Special attention should be addressed to the youngest, those at risk for severe COVID-19 and those with government-imposed work restrictions.

**Supplementary Information:**

The online version contains supplementary material available at 10.1186/s12875-021-01532-7.

## Introduction

During the first outbreak of the COVID-19 pandemic, governments all over the globe had to restrict activities of daily life to avoid the further spread of the disease [1–3]. In Switzerland and other countries, the authorities requested the population to follow hygiene and physical distancing rules. On March 16th, 2020, the Swiss federal government declared a state of emergency due to COVID-19 and imposed severe disease control measures, including the closure of schools, universities, shops, and other businesses, as well as sports, cultural events, and club activities [4]. This lockdown lasted until the withdrawal of the first measures on April 27th. During the lockdown period, up to 17 new cases of SARS-CoV-2 per 100,000 inhabitants were reported each day in Switzerland [5]. Because of this high incidence, it was impossible to test each suspected case and trace their contacts. Persons with respiratory symptoms or fever were advised to undergo a 10-day self-isolation period, and those who had had contact with someone with a positive test for SARS-CoV-2 were strictly recommended to undergo a 10-day period of self-quarantine [6].

Measures to control disease affect daily life, and impact the population’s mental health state [7]. Research shows that pandemics can lead to high levels of stress resulting in mental health problems, such as anxiety, symptoms of depression, insomnia, denial, anger, and fear [8], affecting the general population, healthcare workers, and clinical populations [9–13]. Quarantine heightens feelings of uncertainty and isolation. These feelings are coupled with efforts to cope with the situation within the restrictions imposed by the quarantine itself [14], which include but are not limited to lack of mobility, change of routines, changes in working patterns, and loss of social support [15]. Furthermore, psychological side effects of quarantine and social distancing may include post-traumatic stress, confusion, anger, and symptoms of depression [16–18]. Projections with Swiss data have estimated 0.205 years of life lost due to psychosocial consequences of the COVID-19 disease control measures [19]. Likewise, stress and depression during quarantine induce changes in diet and physical activity, which can have a detrimental impact on physical health [20]. In particular, an unhealthy lifestyle coupled with anxiety increases the risk of long-term cardiovascular disease [20, 21].

Collecting high-quality data on the mental health effects driven by the COVID-19 pandemic was outlined in a recently published position paper by Holmes and colleagues as an immediate priority for mental health research in response to the pandemic [22]. Reliable evidence on the mental health effects of the pandemic is paramount to understand the population’s needs and inform current and future interventions to support mental well-being (particularly in vulnerable groups). These interventions would include all attempts to improve mental health, like population-level policy, occupational health guidelines, and psychological interventions [22].

Thus, the current study aimed to explore how the COVID-19 pandemic broadly affected the mental well-being of the general population in Switzerland. We were interested in well-being as the complete and broad concept of mental health, to which everyone could relate. The World Health Organization defines mental health as an integral part of health, characterised by a state of well-being, in which the person can realize his or her abilities, cope with the stresses of life, be productive and contribute to his or her community [23]. More specifically, and as defined in the literature ‘*well-being refers to how people experience and evaluate their lives and specific domains and activities in their live’* [24]. Usually, the concept of *well-being* relates to health and how well a person’s life is going for that particular person [25]. Therefore, mental well-being is paramount for the complete state of health of the individual and the community. Further, we were interested in assessing demographic, health, and work-related factors associated with impaired mental well-being to better understand which sub-groups of the general population were most affected by the pandemic. This information would be useful for physicians and policymakers to know to which groups would be worth to focus their attention and explore mental well-being, with the aim to improve this aspect of public health during the pandemic and beyond.

## Methods

### Sample selection and data collection

Between March and April 2020, the LINK Institute Lucerne, Switzerland (https://www.link.ch) invited 4110 persons to participate in the Web-based survey to acquire 1000 complete and valid surveys. For 1000 interviews, the sampling error at a 95% confidence interval lies within +/− 3.2% for a percentage of 50% reporting “yes” in a yes/no poll. This sample size enables meaningful analyses between different socio-demographic groups.

Participants were part of the LINK online panel consisting of 115,000 active members. All participants regularly take part in studies, and when a contact is incorrect or ineligible it is removed. No self-selection is possible in the panel, as participants cannot register themselves in the LINK Institute, but the Institute contacts them specifically. The consent to participate in surveys is signed when participants declare themselves available and part of the online panel. Also, they consent when they choose to complete each specific survey.

Participants were selected with quota sampling. They were stratified according to population data from the Federal Statistical Office [26] with quotas based on sex, age (18 to 29, 30 to 44, 45 to 59, and 60 to 79 years old), and language region (German, French and Italian speaking). For example, quota 1 included men between 18 and 29 years old from the German-speaking region. Quota 2 included men between 30 and 44 years old from the German-speaking region, and so on and so forth. Within each quota participants selection was made at random. Additionally, to collect enough interviews per quota, each one was oversampled by 5%.

### Demographic weighting

After data collection, random iterative method (Rim) weighting [27, 28], also known as raking [29], was applied to adjust the survey sample weights to the population for age, sex and language region. This correction adjusts the weights on the specified characteristics until they matched the corresponding proportions (marginal totals) of the population, therefore ensuring that the sample was representative of the general population. This iterative weighting method is efficient for adjusting a survey sample for more than two variables simultaneously with their interlocking quotas. It starts adjusting one variable and then uses the previous adjusted results as the initial values for the next iteration. The process repeats until results converge [30].

### Survey design

The study was an observational, population-based, nationwide, cross-sectional online survey.

Potential participants were sent an invitation by email with the link to the online survey. The invitation specified that if they were part of the target population (as assessed by a few questions at the beginning of the survey), the survey would take about 5 min to complete.

Upon accessing the survey, programmed with IBM® SPSS® Data Collection V7.0 [31], participants read about the department running the survey and the topic (health during the COVID-19 pandemic). Then, they were reminded about data protection and that the data was collected anonymously. By clicking, they agreed to participate in the study, and the survey began.

The questionnaire was fully structured with semi-open and closed questions (see Supplementary Materials). Where relevant, the question had a time reference to the last 2 months (corresponding to the first COVID-19 pandemic wave in Switzerland). Alternatively, some questions referred explicitly to the COVID-19 situation.

#### Mental well-being questions

Participants responded to “Does the current COVID-19 situation impair your mental/emotional well-being?”. This question was naturally worded and easy to understand. It was developed given the lack of standardized tools to assess subjective mental well-being during this pandemic. Alongside, participants also responded to questions relating to specific mental health concerns adapted from two mental health screening tools. From the Patient Health Questionnaire-2 [32, 33] we used the questions a) feeling down or depressed, b) having less interest or pleasure in doing things. The Patient Health Questionnaire-2 was developed to assess the frequency of depressed mood and anhedonia over the past 2 weeks as a first screening approach for depression. Scoring of this questionnaire ranges from 0 to 6 (each question is scored from 0 to 3 based in how frequent the person experiences the symptom, and the total score is the sum of both questions). When the person scores 3 or more it is recommended to follow up with a complete evaluation to assess the possible presence of depressive disorder. From the Generalized Anxiety Disorder-2 questionnaire [34] we developed the item: c) increased worries or feeling more anxious. Originally, the Generalized Anxiety Disorder-2 item has two questions. Scoring ranges from 0 to 6 (0 to 3 for each question, and total score the sum of both). This 2-item questionnaire is used as a first screening tool for generalized anxiety disorder. With a result of 3 or more it is recommended to evaluate the presence of a generalized anxiety disorder. Further, we also asked about d) increased feelings of loneliness. These questions referred to the period spanning the last 2 months. Respondents who indicated the presence of any of the four previous symptoms were then asked: “Did you get advice from a psychologist or physician for your mental health problems?” (yes/no).

#### General health questions

Respondents reported if they had health problems with the question: “Have you had any health problems in the last two months? These include serious illnesses but also minor illnesses, such as headaches, a cold or hay-fever” (possible answers: one health problem, more than one health problem, no health problems). They also responded to specific COVID-19 related questions: 1) if they were part of the population at risk for severe COVID-19 symptoms, as defined by the Swiss Federal Office of Public Health (age over 65 years, patients with cancer, high blood pressure, diabetes, cardiovascular disease, respiratory disease, and diseases that weaken the immune system [35], 2) if someone in their household was part of the population at risk for severe COVID-19, 3) if someone in their household had been in self-quarantine or self-isolation, or 4) if they were tested for COVID-19.

#### Demographics and employment questions

Regarding their employment situation, participants indicated whether they had to stop going to work due to COVID-19. If they had to stop going to work in-person, they chose the reason among the following options. a) they had to work from home, b) they had to stop working because the workplace was closed due to COVID-19 disease control measures (e.g., bars, restaurants, hairdressers, etc.), and it was not possible to work from home, c) they had to stop working because of childcare obligations due to closed day-care centres, d) they had to stop working for other reasons.

Demographic characteristics encompassed age, sex, smoking, household size (1,2, 3 or more people), residential environment (urban or rural), employment status (full-time employment, part-time employment or unemployed), and language region (German, French, or Italian).

### Analyses

All analyses presented were performed on the weighted data in R [36]. The R package “survey” was used to analyse weighted data [37]. The functions of this R package perform weighted estimations, with each observation being weighted by the inverse of its sampling probability. To compute weighted cross-tabulations, we used the “svytable” function. The standard error of proportions was estimated using the functions “svytotal” or “svymean”. Confidence intervals for proportions were calculated using the R function svyciprop with the “likelihood” method which uses the (Rao-Scott) scaled Chi-squared distribution for the loglikelihood from a binomial distribution. We tested the association between impaired mental well-being due to COVID-19 (binary outcome, 1 = impaired, 0 = not impaired mental well-being) and demographic variables (age, sex, household size, urban/rural residential environment, employment status, language region), COVID-19 risk factors (smoking, being oneself or a family member part of the risk group for COVID-19, being in self-quarantine or self-isolation), mental health concerns (feeling lonely, worried or anxious, feeling down or depressed, feeling less pleasure in doing things than before), and changes at work due to COVID-19. For this purpose, we fitted univariable and multivariable generalised linear models of the binomial family to the data with inverse-probability weighting and design-based standard errors using the function “svyglm”. To check for multicollinearity, we performed a bivariate correlation matrix with all covariates using the function “hetcor” from the R package “polycor”. Variance-inflation factors (VIF) for generalized linear models were calculated using the function “VIF” of the R package “DescTools”. The highest VIF value for the model is indicated in the table legend.

## Results

### Weighting

Demographic respondent weightings ranged between a minimum weight of 0.82, and a maximum of 1.36, with 99.8% efficiency, values indicative of a well-balanced and reliable sample [38, 39].

### Participants

From 4110 initially contacted people, 1022 (24.9%) completed the survey. The median age of the weighted sample was 44 years (IQR = 33 to 58, range = 18 to78), and 51% were male. Per regions, 72% were German-speaking, 24% French-speaking, and 4% Italian-speaking respondents. Table 1 shows the demographic characteristics of the sample. Regarding employment, 75% were employed, and among them 47% stopped going to work, mainly because it was recommended that they work from home (27%) or because their workplace was closed due to COVID-19 (14%, see Table 2).

**Table 1:** Demographic characteristics of participants across total sample (N = 1022) and employed population (*n* = 765)

Demographic characteristics	N	*Overall sample (N = 1022)*		
		%	Lower 95% CI	Upper 95% CI
**Sex**				
Women	501	49	46	52
Men	521	51	48	54
**Age group**				
Age (mean)	1022	46	45	47
18–29 years	213	20.8	18	23
30–44 years	298	29.1	26	32
45–59 years	306	29.9	27	33
60–79 years	206	20.1	18	23
**Language region**				
German-speaking	736	72	69	75
Italian-speaking	41	4	3	5
French-speaking	245	24	21	27
**Household size**				
1 person	190	18.7	16	21
2 people	364	35.7	33	39
3 people or more	466	45.7	43	49
**Area type**				
Rural	223	21.8	19	24
Urban	799	78.2	76	81
**Employment**				
Full-time employment	486	47.8	45	51
Part-time employment	276	27.1	25	30
Unemployed	255	25.1	23	28

*N* Total number of participants, % Percentage, *CI* Confidence interval. The numbers are based on the weighted sample. Missing values were found in household size (*n* = 2), employment (*n* = 5)

**Table 2:** Prevalence of population at risk of severe COVID-19, impaired mental well-being and, change in employment situation due to COVID-19, across all participants (N = 1022) and employed population (n = 765)

	N	*Overall sample (n = 1022)*		
		%	Lower 95% CI	Upper 95% CI
**Population at risk for severe COVID-19**				
Smoking	173	17	15	19
Someone in the household in the risk group for COVID-19	264	26	23	29
Are you part of the risk group for COVID-19	260	26	23	28
Are you OR is someone in your household in the risk group for COVID-19	391	38	35	41
Someone In the household in self-quarantine	50	5	4	6
Someone in the household in self-isolation	29	3	2	4
Someone in the household in self-isolation OR in self-quarantine	59	6	5	7
Did you have a COVID-19 test	54	5	4	7
COVID-19 positive test	6	1	0.2	1
**Mental health**				
COVID-19 pandemic impaired mental well-being	347	34	31	37
Felt lonely	242	24	21	26
Felt worried or anxious	298	29	26	32
Felt down or depressed	284	28	25	31
Had less interest or pleasure in doing things	293	29	26	32
Sought advice from a psychologist or physician (total *n* = 570) *	45	8	6	10
Felt lonely OR felt worried or anxious OR felt down or depressed OR had less interest or pleasure in doing things	570	56	53	59
**Employed population (n = 765)**				
Had to stay away from work or stopped working due to COVID-19	479	47	43	50
… because had to work at home	274	27	24	30
… because of closed workplace due to COVID-19	140	14	11	16
… because of other reasons	77	8	6	10
… because had to care for children due to closed day-care centre/school	38	4	3	5
…because of self-isolation due to symptoms	28	3	2	4
… because of self-quarantine due to close contact with suspected or confirmed COVID-19 case	16	2	1	3
… because tested positive for COVID-19	4	0.4	0.1	1

*N* Total number of participants, % Percentage, *CI* Confidence interval. The numbers are based on the weighted sample. Missing values were found in the variable “are you or is someone in your household in the risk group for COVID-19?” (n = 7), “smoking” (n = 2), “someone in the household in self-isolation” (n = 1) or “in self-quarantine” (n = 1), The question “Did you get advice from a psychologist or physician for your mental health problems?” was only posed to those participants who responded “yes” to any of the mental health symptoms. The values for this variable are therefore adapted to an n of 570 individuals instead of the total N of 1022

### How many people were affected directly or indirectly by COVID-19?

Regarding the population at risk, 391 people or 38% of respondents or their co-habitants were at risk of severe COVID-19 symptoms (Table 2). However, only 59 people, or 5.9%, had been in self-isolation or self-quarantine due to COVID-19. Six respondents had a positive test result for COVID-19.

### Impaired mental well-being due to COVID-19

Thirty-four percent of the interviewed population reported that the pandemic impaired their mental well-being. Further screening questions for mental health concerns revealed that 56% of the whole population showed some signs of impaired mental health (feeling lonely, feeling worried or anxious, feeling down or depressed, or feeling less pleasure in doing things than before, see Table 2). Respondents reporting impaired well-being due to the COVID-19 pandemic were significantly more likely to additionally report mental health concerns than respondents not reporting impaired mental well-being (Chi-square test = 350, *P* <  0.001, see Table 1 in Supplementary materials). In those with impaired well-being due to COVID-19, 57% felt anxious or worried, 49% felt lonely, 62% felt down or depressed, and 60% lost interest or pleasure in doing things. In those without impaired well-being due to COVID-19, 14% felt anxious or worried, 13% lost interest or pleasure in doing things, 10% felt lonely, and 10% felt down or depressed. Considering those respondents who reported at least one of the four mental health concerns 8% (45 out of 570 people) contacted a psychologist or physician due to their mental health problems.

### Association of mental health with demographic variables, COVID-19 risk factors, and work situation

**Fig. 1:**
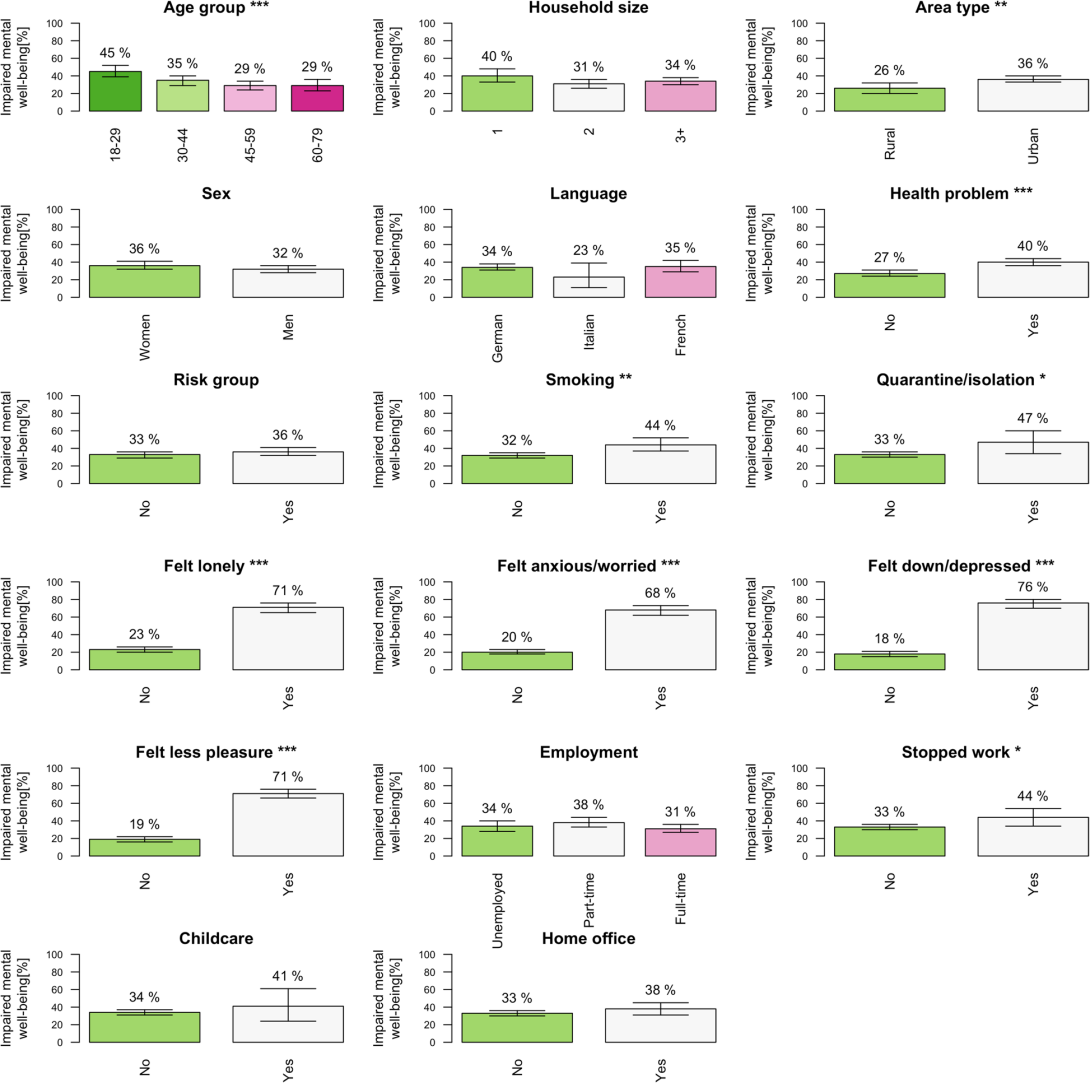
Proportion of people reporting impaired mental well-being due to the COVID-19 pandemic across demographic, health and work-related factors for all participants (*N* = 1022). Legend: * = *P*-value < 0.05, ** = *P*-value < 0.01, *** = *P*-value < 0.001. Stopped work = those who stopped work due to imposed disease control measures, Childcare = those who stopped working to care for their children

Figure 1 presents the proportion of impaired mental well-being across demographic characteristics, COVID-19 risk factors, mental health, and employment-related variables. Table 3 shows the results of a multivariable regression model explaining impaired mental well-being by demographic, health, and employment variables. The likelihood of reporting impaired mental well-being due to COVID-19 was highest in the young, in urban dwellers (vs. rural), in people with health problems (several health problems vs. none), in people being or living with someone at risk of severe COVID-19, in smokers, and people who had to stop working because their workplace was closed due to official disease control measures. In comparison, characteristics associated with a smaller likelihood to report impaired well-being include older age or living in a household of two or more people (compared to living alone).

**Table 3:** Mental well-being status explained by demographic, health, and employment variables

	Multivariable adjusted results					Unadjusted results				
	OR	Lower 95% CI	Upper 95% CI	***P*** value	overall ***P*** value	OR	Lower 95% CI	Upper 95% CI	***P*** value	overall ***P*** value
Age: 18–29 vs. 45–59	1.99	1.32	3.01	0.001	0.002	2.05	1.42	2.96	< 0.001	0.001
Age: 30–44 vs. 45–59	1.3	0.9	1.88	0.17		1.31	0.93	1.86	0.125	
Age: 60–79 vs. 45–59	0.86	0.49	1.51	0.599		1.01	0.67	1.51	0.964	
Household size: 2 vs 1 person	0.65	0.44	0.97	0.036	0.026	0.66	0.45	0.95	0.026	0.029
Household size: 3 or more vs 1 person	0.67	0.45	1	0.052		0.76	0.53	1.08	0.121	
Residency: Urban vs. rural	1.62	1.13	2.32	0.008	0.008	1.66	1.19	2.33	0.003	
Sex: men vs. Women	0.95	0.7	1.29	0.737	0.731	0.82	0.63	1.07	0.142	
Language Region: Italian vs. German	0.49	0.22	1.08	0.079	0.19	0.58	0.27	1.25	0.167	0.316
Language Region: French vs. German	0.92	0.66	1.28	0.629		1.06	0.78	1.43	0.733	
Health problems (One vs. none)	1.31	0.95	1.8	0.102	0.005	1.54	1.14	2.07	0.005	< 0.001
Health problems (several vs. none)	1.88	1.29	2.74	0.001		2.28	1.61	3.24	< 0.001	
Being or living with someone at risk for severe COVID-19	1.38	1	1.9	0.051	0.001	1.18	0.9	1.55	0.22	
Smoking	1.8	1.24	2.61	0.002	0.001	1.68	1.2	2.36	0.003	
Someone in the household was in self-quarantine or self-isolation	1.57	0.92	2.66	0.097	0.08	1.76	1.04	2.97	0.035	
Couldn’t work due to closed workplace	1.66	1.04	2.67	0.035	0.038	1.61	1.06	2.45	0.026	
Couldn’t work due to childcare	1.47	0.66	3.24	0.345	0.35	1.39	0.65	2.94	0.395	
Home office	1.23	0.86	1.76	0.258	0.261	1.25	0.91	1.72	0.173	
Employed part time	1.02	0.63	1.64	0.936	0.079	1.19	0.83	1.71	0.337	0.019
Employed full time	0.8	0.5	1.28	0.349		0.88	0.64	1.22	0.453	

Adjusted analysis (left side): all variables are corrected for all the other variables in the table. The highest variance inflation factor was 1.64 for employment status. Unadjusted analyses (right side). *OR* Odds ratio, *CI* Confidence Interval. The variable “sought advice from a psychologist or physician” was excluded from the model because only 570 participants had the chance to respond to it (those reporting at least one mental health concern)

Table 4 presents a multivariable regression model including all independent variables from the previous model and the four mental health concerns. The likelihood of impaired mental well-being was most likely in those respondents being or living with someone at risk for severe COVID-19 symptoms and those reporting any of the four mental health concerns (feeling lonely, feeling worried or anxious, feeling down or depressed, or feeling less pleasure in doing things).

**Table 4:** Mental well-being status explained by demographic and mental health concerns variables

	Multivariable adjusted results				Unadjusted results			
	OR	Lower 95% CI	Upper 95% CI	***P*** value	OR	Lower 95% CI	Upper 95% CI	***P*** value
**Felt lonely** (yes vs. no)	4.08	2.53	6.58	< 0.001	8.35	6.02	11.59	< 0.001
**Felt anxious or worried** (yes vs. no)	6.13	3.91	9.59	< 0.001	8.19	6.03	11.12	< 0.001
**Felt down or depressed** (yes vs. no)	7.21	4.5	11.55	< 0.001	14.15	10.16	19.71	< 0.001
**Felt less pleasure in doing things** (yes vs. no)	6.28	4.1	9.62	< 0.001	10.49	7.65	14.38	< 0.001

Adjusted analysis (left side): all variables adjusted for variables presented in Table 3. The highest variance inflation factor was 1.98 for employment status. Unadjusted analyses (right side). *OR* Odds ratio, *CI* Confidence Interval

## Discussion

This study highlighted how the first wave of the COVID-19 pandemic broadly impaired the mental well-being of the general Swiss population. Precisely, one-third (34%) of respondents reported impaired mental well-being. Variables associated with impaired mental well-being were being young, being at risk for a severe outcome, having one or more health problems, smoking, living in a single household, and stopping work due to disease control measures.

Our finding is coherent with another study conducted in Switzerland by de Quervain and colleagues focusing on stress. Their study collected data at two time points: the beginning of lockdown and during the partial lifting of lockdown measures. They focused on changes in stress levels and symptoms of depression. The comparative time point between the two studies is the beginning of lockdown. They found that about half of their sample (49.6%) had increased stress during confinement compared to stress levels before the COVID-19 pandemic. Respondents also reported a 57% increase in symptoms of depression, which were highly correlated with the changes in stress. The authors identified several reasons for the increased stress levels, such as changes at work, problems with childcare, or not being able to spend time with others [40]. In our study, we also found a significant association with increased impaired mental well-being in those respondents who could not go to work because the workplace was closed as mandated by the government. Our results are also comparable with findings from the UK, Spain, and Italy during the first wave lockdown (between April 24th and May 1st). There, around 43% of the population was expected to be at risk of stress, anxiety, and depression [41]. Perhaps because of the stricter lockdown measures imposed on those countries, the proportion of impaired mental health was higher. For example, Spain had hard confinement with internal travel restrictions, and with strict stay-at-home requirements. The population was allowed to leave home only to go to the grocery store, the doctor or the pharmacy. These restrictions were also valid for children. Meanwhile, in Switzerland stay at home was also expected but was not enforced and remained a recommendation from the authorities appealing to the citizens’ sense of personal responsibility. Likewise, it was recommended to self-isolate and self-quarantine as necessary. Additionally, closures of public spaces and restrictions to freedom of movement were the responsibility of the Cantons, and the Federal Government did not impose a general rule for the whole country [4, 42–44]. We were further able to highlight the characteristics of those respondents who self-rated their mental well-being as impaired.

### Demographic covariates of impaired mental well-being: age, residential environment and, household size

Older people had a smaller likelihood to report impaired mental well-being due to COVID-19 than younger people. In comparison, people aged between 18 and 29 had a doubling in the odds of reporting impaired mental well-being than respondents between 45 and 59 years old (OR = 1.99, *P* = 0.001). This finding is consistent with the literature showing older age as a resilience factor for mental well-being [40, 45–48] and younger age as a predictor for poor mental health and well-being [49, 50]. According to Pro Juventute, a charitable foundation in Switzerland, its helpline has registered increasing demand from young people. Psychological consultations increased 40% between October and December 2020 compared to the same period last year. Their survey indicated that nearly 60% of 15–34-year-olds felt isolated and alone in society, more than any other age group [51].

Regarding the type of residential environment, we found that urban living was associated with a 62% increase in the odds of reporting impaired mental well-being (OR = 1.62, *P* = 0.008) compared to living in a rural environment. The environmental and social conditions of urban areas might challenge mental well-being. On the one hand, urban areas provide more opportunities for socializing, education, culture, work, and easier access to health care. On the other hand, urban-living includes easier access to drugs, exposure to crime and violence, poverty, pollution, traffic, loneliness, and a consequent higher need for stress processing [52, 53]. To these life challenges occurring during everyday life, we have to add the restrictions imposed by the government during the lockdown and the higher risk of SARS-CoV-2 infection [54].

We also found that respondents living with more people reported less impaired mental well-being than those living in single households. For example, respondents living in a household of two people had a 35% lower odds of reporting impaired mental well-being than those living alone (OR = 0.65, *P* = 0.036). This protective effect of living with more people corresponds to other studies showing a potential association between living alone and low positive mental health (defined as comprising both hedonic and eudaimonic elements of mental wellbeing) as shown in a systematic review of studies published between 2014 and 2017 [55]. During the pandemic, this might be especially important as people might fear spending the period of self-quarantine or self-isolation alone at home [56].

### Health covariates of impaired mental well-being: risk for severe COVID-19, smoking, and health problems

Our study showed that participants at risk of severe COVID-19 or sharing their household with someone at risk for severe COVID-19, had a 38% higher odds of reporting impaired mental well-being than participants not in this situation (OR = 1.38, *P* = 0.05). These results correspond with literature showing chronic medical conditions as risk factors for anxiety and depression [57, 58], and therefore impaired mental well-being.

Regarding smoking, our results are in line with other studies that show that smoking is associated with poor mental health [49]. Smokers in our study were more likely to report impaired mental well-being than non-smokers (OR = 1.8, *P* = 0.002). There is some contradictory evidence in the literature regarding the effect of smoking on COVID-19 infection severity. A preliminary meta-analysis suggested that active smoking was not significantly associated with the severe progression of COVID-19 [59]. Nevertheless, increasing evidence indicates that smoking is more prevalent among severe cases of COVID-19 and probably also COVID-19 related deaths [60]. In addition, risk factors for a severe COVID-19 outcome are often present in smokers, i.e., lung and cardiovascular disorders [61].

We found that respondents with more health problems were more likely to report impaired mental well-being than their counterparts with no health problems (OR = 1.88, *P* = 0.001). This was expected, as physical and mental health are both considered by the World Health Organization (WHO) as integral dimensions of health and well-being [62], and they are dynamically related [63, 64].

### Employment-related covariates of impaired mental well-being

Considering only the employed population, respondents who had to stop going to work due to a closed workplace had a 66% increased odds of reporting impaired mental well-being than other employed respondents who could continue going to work (OR = 1.66, *P* = 0.035). Beyond the physical infection, the pandemic brings along also economic effects derived from changes in the work situation that might trigger worries about losing one’s job, and therefore falling into a challenging personal financial situation and ultimately contributing to higher income inequality in society. Evidence suggests that this inequality affects population health and well-being and that this is most likely mediated through psychological stress (which leads to impaired general health and depression) [65, 66].

### Access to mental health interventions and contact restrictions

Access to mental health care was diminished due to the COVID-19 pandemic disruption of services around the world. Factors that affect mental health services include, among others, risk of infection in long-stay institutions, barriers to meeting in-person or even reduction of available mental health professionals due to infection [67]. Given the need to reduce personal interactions, online mental health services start to be widely accepted internationally [12] and are well suited to providing access to health services without carrying any risk of infection [68]. In Switzerland, psychiatric and psychological ambulatory care providers seem to have maintained the same level of service during the lockdown as before the pandemic, thanks to the government measures to finance teleconsultations [69]. Remote sessions allowed service-provision while complying with the need for physical distancing. Simultaneously, there was an increase in demand for psychological support, especially from the younger citizens [70], with some services reporting a 12% increase in calls compared to the previous year [71]. Furthermore, we need to consider that depending on the evolution of the pandemic (i.e., duration and economic consequences), it is possible that still more people than usual will need psychosocial support, and this support would be most efficient when delivered through different channels. As reported by the Swiss Federal Office of Public Health, different generations, for example, tend to seek information and support in different ways: older residents tend to use the phone, the internet is mostly used by those in their middle age, and the younger tend to prefer mobile apps [72], chat, email or SMS [69].

### Promotion of mental well-being

Depending on how strict the governmental lockdown measures are, the promotion and support of mental health during a pandemic might become necessary. Strategies to cover pandemic-related mental health difficulties include the long-term maintenance of helplines for mental health support (in Switzerland currently financed by the Federal Office of Public Health) and the creation of new ones. New helplines could be advertised through several media (radio, TV, social media) to reach all population segments. Other strategies could involve increasing the support offered by psychiatric and psychotherapeutic care entities, especially as patients with previous mental illnesses are vulnerable to further impairment of their well-being during the pandemic. Besides, also mental health care could be further supported by general practitioners and pediatricians. Our findings suggest that public health initiatives providing social support and information about where to get help and remain connected (e.g., via helplines) should target particularly young people, people at risk for severe COVID-19, and those with an insecure financial situation as a result of the lockdown. As suggested by Gloster et al., [73], interventions that promote psychological flexibility may alleviate the negative mental health consequences of the pandemic. Gloster et al., exemplify psychological flexibility as holding one’s thoughts lightly, be accepting of one’s experiences, engage in what is important to one despite challenging situations. On the economic level, those whose finances have worsened because of the pandemic measures should get quick and uncomplicated financial aid.

### Limitations

The study was conducted in Switzerland and given cultural differences among countries, and the reactions of their governments to the pandemic, results might not be generalisable to populations other than the Swiss one. Within Switzerland, we also found differences among language regions. Residents from the Italian-speaking region reported significantly less impaired mental well-being than those from the German-speaking region. This result seems to reflect the cultural differences between regions in Switzerland, which we wanted to control for.

Also, the cross-sectional nature of the study has certain limitations. To be able to generalise results to the population the study sample has to be representative. For a population of about 8 million people and a survey sample of 1022 individuals, sampled at random per quotas from a very extensive panel, we estimated a margin of error of +/− 3.2% (for questions with two possible answers). Further, results were weighted based on age, sex, and language-speaking region data published by the Federal Office of Public Health, therefore ensuring that the sample was representative of the general population of Switzerland. Also, it is to be acknowledged that a cross-sectional design does not allow us to establish causal relationships, and thus our results show the strength of associations.

This study focused on self-reported impaired well-being during the lockdown during March and April 2020, using a simple question formulated to be understood by everyone. However, this question has not yet been validated. To validate this question it would be interesting to investigate objective measures of well-being, during the same period or even long-term, given that psychological effects after a pandemic tend to last long-term [74, 75]. Nonetheless, we found a strong association between impaired mental well-being and the mental health concerns variables from validated instruments (namely feeling lonely, feeling worried or anxious, feeling down or depressed, or feeling less pleasure in doing things). This strong association (see Supplementary materials) suggests that the question about “impaired mental well-being” measured what we intended. It might have worked as a general well-being concept which included the emotional components of the four mental health concerns.

We highlight here the possibility that our question “*Does the current COVID-19 situation impair your mental/emotional well-being?* might feel leading to some readers. In hindsight, we think that it would have been appropriate to build this question in a more neutral way. For example, avoiding the word ‘impair’ directly in the question and perhaps rephrasing it as ‘influenced in any direction your mental well-being’. Consequently, we cannot completely exclude the possibility that our results might be biased given the structure of the question. Nevertheless, we do not think this is the case for the following reasons. (1) The COVID-19 pandemic, specially the first wave, had such an unprecedented and important impact in peoples’ lives, that we do not think anyone would be easily led by our question. On the one hand some people had their mental well-being challenged, while others felt a relieve from their daily habits. (2) The cross-tabulation between mental well-being and the screening questions (felt lonely, anxious, depressed, had less pleasure in doing things than before), shows significant differences in mental well-being between the group of participants who reported impaired mental well-being (who had significantly more symptoms) and the group reporting not-impaired mental well-being (less symptoms). This significant relationship between the variables is evidence that our main question was measuring mental well-being. It would have been suspicious if there were no significant differences between the groups. (3) Other studies, measuring different aspects of mental well-being found similar proportions of people affected [40, 41]. If our question would have been leading, then we can imagine that our results would have been inflated and a much higher proportion of the population would have reported impaired mental well-being.

Also, it would have been interesting to study the link between local area deprivation and mental well-being status. Local area deprivation indices measure certain socioeconomic conditions, including social and material disadvantages [76] in relatively small geographical areas [77]. Deprivation indices are usually associated with health outcomes [78] and therefore interesting to associate with measures of well-being. We could not calculate such an index with the current dataset, nor are we aware of such an index validated for Switzerland. Also, we did not control for other economic factors like respondent’s salary or household total income. Other studies in Switzerland have shown that there is an effect of economic deprivation in psychological well-being [79], and others have found that participants with a lower socio-economic profile have a low participation rate in health surveys [80]. Thus, we cannot exclude the possibility that our results are slightly biased and that the real impact of the first wave of COVID-19 pandemic was indeed even more challenging for the population.

It would have been interesting to ask those two-thirds of respondents who did not report impaired mental well-being, how they felt. Other studies have shown some people remained neutral, while for others the pandemic brought a situation to stress relief compared to their lives before the pandemic [40]. Given the importance of resilience, it would have also been interesting to ask about factors that people consider useful to keep physically and mentally healthy while facing stressful life events.

## Conclusion

We studied self-reported mental well-being status during the first wave of the COVID-19 pandemic in a representative sample of the Swiss population. Impaired mental well-being was associated with younger age, urban residential environment, single household, health problems, being at risk for severe COVID-19 symptoms, smoking, and not being able to go to work because the workplace was closed as imposed by the government. Given the current figures on the effect of COVID-19 on mental well-being in the Swiss population, it seems necessary to increase the focus on mental well-being in the young and people at risk for COVID-19. Given the possible long-term consequences of impaired mental well-being, it is in the best interest of public health to promote and increase measures to improve the mental health of those populations. We, therefore, encourage policymakers to keep developing, funding, and implementing strategies for mental health assessment, support, treatment, and promotion during this extraordinary pandemic situation.

## Supplementary Information

**Additional file 1 **Questionnaire in English. **Table S1** Cross-tabulation of mental health well-being reported impairment due to COVID-19 and screening questions of impaired mental health.

## Data Availability

The datasets used and/or analysed during the current study are available from the corresponding author on reasonable request.
