# Supplementary material for: The effect of COVID-19 on mental well-being in Switzerland: a cross-sectional survey of the adult Swiss general population
Source: BMC Fam Pract. 2021 Sep 10;22:181. doi: 10.1186/s12875-021-01532-7 (PMC8432273; doi:10.1186/s12875-021-01532-7)
Supplement: Supplementary file 1 — Additional file 1 Questionnaire in English. Table S1 Cross-tabulation of mental health well-being reported impairment due to COVID-19 and screening questions of impaired mental health. [file 12875_2021_1532_MOESM1_ESM.docx]

# Supplementary materials

## Questionnaire in English

All questions made explicit reference to the last two months or to the COVID-19 pandemic situation.

| Population at risk for severe COVID-19 |
| --- |
| Have you had any health problems in the last two months? These include serious illnesses but also minor illnesses, such as headaches, a cold or hay fever (one health problem, more than one health problem, no health problems) (yes/no) |
| There are "people particularly at risk" for a severe course of coronavirus infection. These include people over 65 years of age, patients with cancer, high blood pressure, diabetes, cardiovascular disease, respiratory disease, and diseases that weaken the immune system.  Do you belong to this risk group? (yes/no) |
| Is someone of your household in the risk group for COVID-19? (yes/no) |
| Do you smoke? (yes/no) |
| Was someone from your household in self-quarantine? (yes/no) |
| Was someone from your household in self-isolation? (yes/no) |
| Employment situation due to COVID-19 |
| Did you have to stay at home/not work due to COVID-19? (yes/no) |
| If yes, why? |
| Because I was tested positive for COVID-19 (yes/no) |
| Because the GP or another information helpline/assistance recommended to stay at home in self-isolation due to my symptoms(yes/no) |
| Because the GP or another information helpline/assistance recommended to stay at home in self-quarantine due to close contact with COVID-19 positive person (yes/no) |
| Because my workspace was closed due to COVID-19(yes/no) |
| Because I could not work because I had to care for my kids(yes/no) |
| Because I had to work at home(yes/no) |
| Because of other reasons(yes/no) |
| Mental well-being |
| Does the current COVID-19 situation impair your mental/emotional well-being? (yes/no) |
| During the last two months weeks, did you suffer any of the following symptoms? |
| I felt lonely (yes/no) |
| I was worrying or feeling more anxious than usual (yes/no) |
| I felt down or depressed (yes/no) |
| Less interest or pleasure in doing things than usual (yes/no) |
| *(If responded yes to any of the symptoms of the previous question, this question followed)*  Did you get advice from a psychologist or physician for your mental health problems? (yes/no) |

## Table S.1: Cross-tabulation of mental health well-being reported impairment due to COVID-19 and screening questions of impaired mental health

|  |  | COVID-19 pandemic impaired mental well-being | |  |  |
| --- | --- | --- | --- | --- | --- |
|  |  | Yes | No | Chi-  squared | *P*-value |
|  |  | N (%) | N (%) |  |  |
| Felt lonely | Yes | 167 (49%) | 69 (10%) | 188 | <0.001 |
|  | No | 173 (51%) | 594 (90%) |  |  |
| Felt anxious or worrying | Yes | 195 (57%) | 93 (14%) | 205 | <0.001 |
|  | No | 146 (43%) | 569 (86%) |  |  |
| Felt down or depressed | Yes | 211 (62%) | 69 (10%) | 301 | <0.001 |
|  | No | 129 (38%) | 594 (90%) |  |  |
| Had less interest or pleasure in doing things | Yes | 206 (60%) | 84 (13%) | 249 | <0.001 |
|  | No | 135 (40%) | 578 (87%) |  |  |
| Compound measure (felt lonely or anxious or depressed or less interested) | Yes | 327(96%) | 228(34%) | 350 | <0.001 |
|  | No | 14 (4%) | 434(65%) |  |  |
